# Draft genome sequence of a *Wolbachia* endosymbiont from *Syringophilopsis turdi* (Fritsch, 1958) (Acari, Syringophilidae)

**DOI:** 10.1128/MRA.00605-23

**Published:** 2023-10-26

**Authors:** Eliza Głowska, Michael Gerth

**Affiliations:** 1 Department of Animal Morphology, Faculty of Biology, Adam Mickiewicz University in Poznań, Poznań, Poland; 2 Department of Zoology, Faculty of Natural Sciences I, Martin Luther University Halle-Wittenberg, Halle (Saale), Germany; 3 German Centre for Integrative Biodiversity Research (iDiv) Halle-Jena-Leipzig, Leipzig, Germany; Indiana University, Bloomington, Indiana, USA

**Keywords:** *Wolbachia*, symbiosis, evolution, mites (Acari)

## Abstract

We present the draft genome of a *Wolbachia* endosymbiont from quill mites. This is the first representative of a recently discovered distinct *Wolbachia* lineage (supergroup P). We hope the genome will be a useful resource for comparative evolutionary and genomic studies across the globally distributed symbiont *Wolbachia*.

## ANNOUNCEMENT

*Wolbachia* is a very common intracellular, maternally transmitted symbiont of arthropods and nematodes (1). The bacteria have profound effects on host reproduction and physiology, as well as on host-pathogen interactions (2). Due to the difficulty of culturing *Wolbachia* in cell-free media, genetically distinct *Wolbachia* lineages are designated as “supergroups” rather than species, and to date, 21 of such supergroups have been identified (3). However, many of these supergroups lack genomic representation, which hinders comparative evolutionary studies. Quill mites (Acari: Syringophilidae) are parasitic mites found in feather quills of many bird species (4). Previously, two *Wolbachia* supergroups not currently known from other hosts were detected in several species of quill mite (5, 6). We here present the first draft genome for one of these lineages, supergroup P, which is most closely related to *Wolbachia* supergroups C and F (5).

Mite material was originally sampled from feathers of Song thrush *Turdus philomelos* Brehm (April 2009, Kopań, Poland) and stored in 96% ethanol. DNA was extracted from a single adult individual of *Syringophilopsis turdi* using the DNeasy Blood & Tissue Kit (Qiagen, Hilden, Germany) by incubating the specimen in lysis buffer at 56°C overnight and digesting it using Proteinase K for 72 h as commonly done for quill mites (7). The intact exoskeleton was then removed from the buffer to be mounted on a microscopic slide and DNA extraction continued using the manufacturer’s protocol. Identification was done using a microscope and the morphological characters described in reference (8). The DNA was stored in elution buffer until sequencing. The library for Illumina sequencing was prepared using a protocol for double-indexed libraries (9, 10). First, DNA was sheared using a Covaris E210 to insert sizes of 300 bp, followed by a blunt end repair using T4 DNA Polymerase, adapter ligation with T4 DNA ligase, and the Illumina adapters P5 and P7. Between each of these steps, the library was purified using SPRI beads. Library preparation success was determined by qPCR, and indices were added in an indexing PCR. The library was sequenced as 96 bp paired-end run on an Illumina HiSeq 2000. We performed base calling with freeIbis using default parameters (11), trimmed adapters, and discarded all reads with more than five bases below a quality threshold of 15. Sequencing resulted in a total of 22.44 M read pairs, which were assembled using metaSPAdes version 3.15.3 (12) under default parameters. *Wolbachia* contigs were identified using BLAST+ searches (13) against the NCBI nt database and subsequent contig filtering by taxonomy (genus: *Wolbachia*), coverage (≥50×), and GC (guanine-cytosine) content (≥0.2 and ≤0.4) with Blobtools version 1.1.1. (14). Out of 119,320 metagenomic contigs, 163 remained after this initial filtering. Paired-end reads aligning to these 163 contigs were then used for a second round of assembly using SPAdes version 3.15.3. (15) and k-mer sizes 21, 33, 55, 77, and 91, and contigs shorter than 500 bp were discarded.

The *w*Stur draft genome contains 14 contigs with an average GC content of 34% and a total length of 1,054,444 bp (N50 = 249,347 bp). The final coverage was 106×. NCBI’s Prokaryotic Genome Annotation Pipeline version 6.5 (16) predicted 1,056 CDS (coding sequences), 34 of which were pseudogenes. The average CDS length is 761 bp, and the genome-wide coding density is 79%. Using the Rickettsiales-specific marker genes, we estimated the genome to be 100% complete with 0.32% contamination by CheckM version 1.1.3. (17). Using BLAST+ searches, we confirmed that the partial 16S sequence reported for the supergroup P *Wolbachia* (5) is 100% identical with the 16S sequence of *w*Stur. Five CDS had similarities to the *Wolbachia* insertion sequences ISWosp8, ISWen3, or ISWpi15 as determined with ISFinder (18) BLAST+ searches (e-value 1e-6). Using the PHASTER web server (19), we were unable to detect any prophage sequences in the draft genome. Overall, these characteristics indicate that *w*Stur is a typical *Wolbachia* genome that has lost most of its mobile genetic elements. The draft genome sequence will be useful for studying *Wolbachia* genome evolution and *Wolbachia*-quill mite interactions.

## Data Availability

This Whole Genome Shotgun project has been deposited in GenBank under the accession no. JAUCRB000000000. The version described in this article is the first version, JAUCRB000000000.1. Illumina reads are available under NCBI SRA accession no. SRR24987314.
